# Supplementary material for: Multi-dimensional optical information acquisition based on a misaligned unipolar barrier photodetector
Source: Nat Commun. 2024 Aug 16;15:7071. doi: 10.1038/s41467-024-51378-7 (PMC11329724; doi:10.1038/s41467-024-51378-7)
Supplement: Supplementary file 1 — Supplementary Information [file 41467_2024_51378_MOESM1_ESM.pdf]

Supplementary Information:

Multi-dimensional optical information acquisition based on a misaligned unipolar barrier photodetector

Shukui Zhang[1,2,6], Hanxue Jiao[1,6], Yan Chen[1,3,*], Ruotong Yin[1], Xinning Huang[1], Qianru Zhao[1], Chong Tan[1], Shenyang Huang[4], Hugen Yan[4], Tie Lin[1], Hong Shen[1], Jun Ge[1], Xiangjian Meng[1], Weida Hu[1,2], Ning Dai[1,2], Xudong Wang[1,*], Junhao Chu[1,3], Jianlu Wang[1,2,3,5,*]

[1]*State Key Laboratory of Infrared Physics, Shanghai Institute of Technical Physics, Chinese Academy of Sciences, 500 Yu Tian Road, Shanghai 200083, China;*

[2]*Hangzhou Institute for Advanced Study, University of Chinese Academy of Sciences, Hangzhou, Zhejiang 310024, China;*

[3]*Institute of Optoelectronics, Shanghai Frontier Base of Intelligent Optoelectronics and Perception, Fudan University, Shanghai 200433, China;*

[4]*State Key Laboratory of Surface Physics and Department of Physics, Fudan University, Shanghai 200438, China.*

[5]*Frontier Institute of Chip and System, Fudan University, Shanghai 200433, China*

[6]*These authors contributed equally: Shukui Zhang, Hanxue Jiao*

[*]Corresponding authors.

Yan Chen: yanchen_@fudan.edu.cn;

Xudong Wang: wxd0130@mail.sitp.ac.cn;

Jianlu Wang: jlwang@mail.sitp.ac.cn

Supplementary Note 1

Transition states and opening voltage V_{op}. Under the condition of zero bias, each junction region generates photogenerated electron-hole pairs under illumination, and the photogenerated electron-hole pairs are separated and collected to form photocurrent under the action of the built-in electric field. Because the E_{in1} and E_{in2} are in opposite directions, the photocurrent formed by junction 1 and junction 2 under their respective internal electric fields is also in opposite directions, as shown in Supplementary Fig. 4e. The polarity of the photocurrent produced in region I and region II is opposite, and the polarity of the current generated in region III is determined by the greater absolute value of the photocurrent generated in junction 1 and junction 2. The photocurrents corresponding to regions I, II, and III are shown below:

$$I_{\mathrm{I}} = -p_1 q \mu_1 E_{in1} A_{\mathrm{I}} \tag{1}$$

$$I_{\mathrm{II}} = p_2 q \mu_2 E_{in2} A_{\mathrm{II}} \tag{2}$$

$$I_{\mathrm{III}} = -p_1 q \mu_1 E_{in1} A_{\mathrm{III}} + p_2 q \mu_2 E_{in2} A_{\mathrm{III}} \tag{3}$$

$$\begin{aligned} I_{\mathrm{total}} &= I_{\mathrm{I}} + I_{\mathrm{II}} + I_{\mathrm{III}} \\ &= -p_1 q \mu_1 E_{in1} \left(A_{\mathrm{I}} + A_{\mathrm{III}}\right) + p_2 q \mu_2 E_{in2} \left(A_{\mathrm{II}} + A_{\mathrm{III}}\right) \end{aligned} \tag{4}$$

Where q is the unit charge, p_1, μ_1, p_2, and μ_2 are the hole concentration and hole mobility of b-AsP and bP, respectively. A_I, A_{II}, and A_{III} are the areas of regions I, II, and III respectively. The current in region III in Supplementary Fig. 4e is negative ($I_{III} < 0$), which means $|p_1\mu_1E_{in1}A_{III}| > |p_2\mu_2E_{in2}A_{III}|$, indicating that the current in region III at this time is dominated by the current generated by b-AsP.

Supplementary Fig. 4a shows the photocurrent mapping when 0.1 V forward bias is applied, the current expression for each region is shown below:

$$I_{\mathrm{I}} = p_1 q \mu_1 (E_{ex} - E_{in1}) A_{\mathrm{I}} \tag{5}$$

$$I_{\mathrm{II}} = p_2 q \mu_2 (E_{ex} + E_{in2}) A_{\mathrm{II}} \tag{6}$$

$$I_{\text{III}} = p_1 q \mu_1 (E_{ex} - E_{in1}) A_{\text{III}} + p_2 q \mu_2 (E_{ex} + E_{in2}) A_{\text{III}} \tag{7}$$

$$\begin{aligned} I_{\text{total}} &= I_{\text{I}} + I_{\text{II}} + I_{\text{III}} \\ &= p_1 q \mu_1 (E_{ex} - E_{in1}) \left(A_{\text{I}} + A_{\text{III}}\right) + p_2 q \mu_2 (E_{ex} + E_{in2}) \left(A_{\text{II}} + A_{\text{III}}\right) \end{aligned} \tag{8}$$

Compared with zero bias, the reverse photocurrent value of region I becomes smaller (maximum value decreased from 2.2 μA to 1.3 μA, as shown in Supplementary Figs. 4e and 4a), while the forward photocurrent of region II becomes larger (maximum value increased from 0.5 μA to 1.1 μA, as shown in Supplementary Figs. 4e and 4a), indicating that junction 1 is suppressed, while junction 2 is enhanced. Since the current in region I is still negative at this time, the value of E_{ex} is less than E_{in1}, indicating that the voltage required to open the BP channel has not reached.

When the bias voltage is gradually applied to $E_{ex} > E_{in1}$, junction 1 is in the OFF state. Under the action of bias voltage, the holes generated by junction 1 should transport to the right, as shown in Fig. 2f. Because of the existence of the hole barrier layer, the electron-hole pairs generated by both photoexcitation and thermal excitation in the b-AsP layer are quickly recombined due to the hole obstruction, which means that the barrier layer can not only reduce the electrical crosstalk but also inhibit the dark current. The current of each junction is shown as follows:

$$I_{\text{I}} = 0 \tag{9}$$

$$I_{\text{II}} = p_2 q \mu_2 (E_{ex} + E_{in2}) A_{\text{II}} \tag{10}$$

$$I_{\text{III}} = p_2 q \mu_2 (E_{ex} + E_{in2}) A_{\text{III}} \tag{11}$$

$$I_{\text{total}} = I_{\text{I}} + I_{\text{II}} + I_{\text{III}} = p_2 q \mu_2 (E_{ex} + E_{in2}) \left(A_{\text{II}} + A_{\text{III}}\right) \tag{12}$$

Only the electron holes generated by the bP layer can be collected to form a current, indicating that the opening working voltage $V_{op\text{-}J1}$ of junction 1 is reached. The operating voltage $V_{op} = E_{ex}d$, where d is the thickness of the heterojunction, since the OFF state condition of junction 1 is $E_{ex} > E_{in1}$, the operating voltage of Junction 2 can

be obtained to meet $V_{op\text{-}J2} > E_{in1}d$. In contrast, the operating voltage of junction 1 must meet the condition $V_{op\text{-}J1} > E_{in2}d$. After many experiments, it has been shown that junction 1 and junction 2 can usually be opened at the reverse bias of -0.4 V and the forward bias of 0.4 V, respectively.

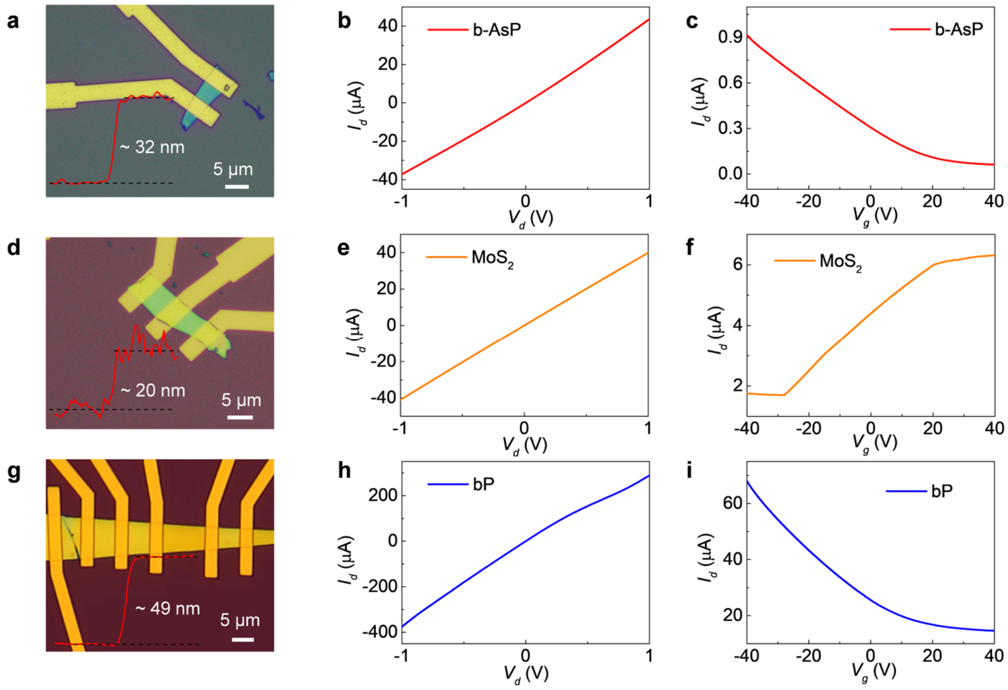

Supplementary Fig. 1 | Transfer and output characteristic curves of b-AsP, MoS_2, and bP field effect transistors (FETs). a-c, Optical photo, material thickness, output characteristic curve and transfer characteristic curve of b-AsP FET. **d-f**, Optical photo, material thickness, output characteristic curve and transfer characteristic curve of MoS_2 FET. **g-i**, Optical photo, material thickness, output characteristic curve and transfer characteristic curve of bP FET.

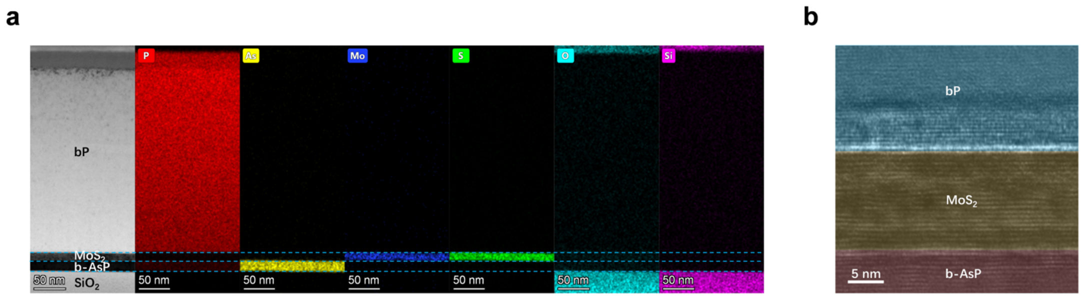

Supplementary Fig. 2 | TEM images of the b-AsP/MoS_2/bP heterojunction. **a**, Cross-sectional TEM image and EDS mapping of the b-AsP/MoS_2/bP heterostructure. Elements P, As, Mo, S, O, and Si are marked with different colors. **b**, High-resolution cross-sectional TEM image of the interface.

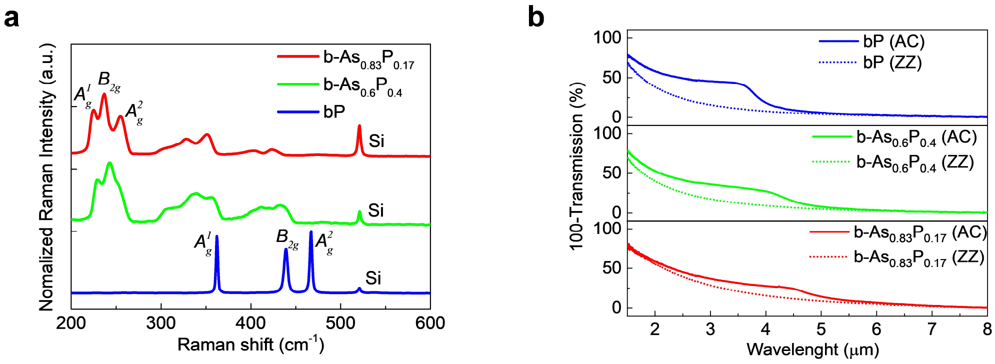

Supplementary Fig. 3 | Materials characterization a, Raman spectra measured for bP, b-$As_{0.6}P_{0.4}$, and b-$As_{0.83}P_{0.17}$. **b**, Polarization transmission spectra of bP, b-$As_{0.6}P_{0.4}$, and b-$As_{0.83}P_{0.17}$.

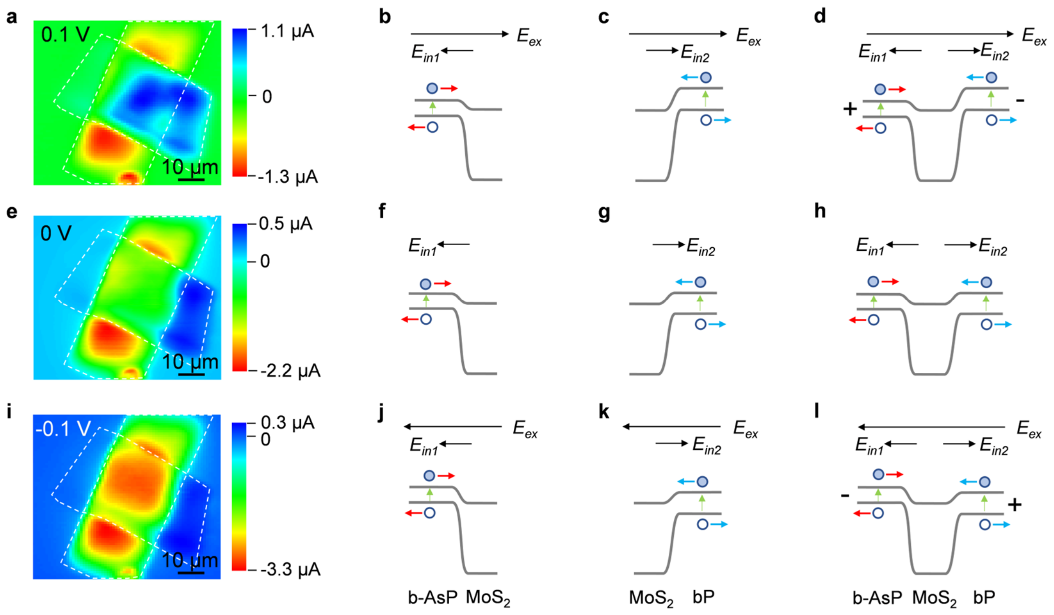

Supplementary Fig. 4 | The transition states before reaching the operating voltage. **a-d**, Photocurrent mapping and energy band diagrams of corresponding regions I, II, and III at a positive bias voltage of 0.1 V. **e-h**, Photocurrent mapping and energy band diagrams of corresponding regions I, II, and III at a bias voltage of 0 V. **i-l**, Photocurrent mapping and energy band diagrams of corresponding regions I, II, and III at a negative bias voltage of - 0.1 V.

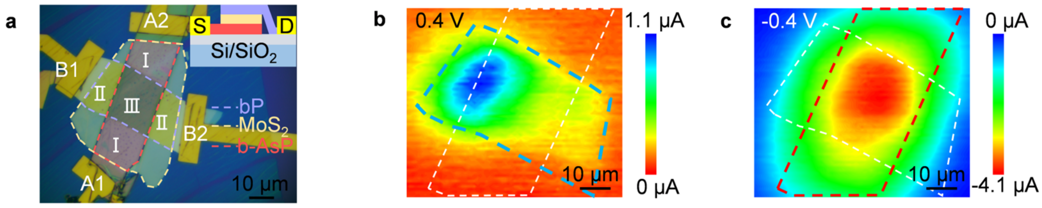

Supplementary Fig. 5 | The voltage-addressing behavior of b-AsP/MoS_2/bP pBp photodetector. a, Optical microscope image of the device. The contours of b-AsP, MoS_2, and bP highlighted by red, orange, and blue dashed lines, respectively. A1, A2, B1, B2 are electrodes. Upper right corner: the diagram of the device structure, with b-AsP (red), MoS_2 (orange), and bP (blue) stacked from bottom to up. **b**, **c**, Photocurrent mapping of the device under forward bias and reverse bias respectively. These measurements were performed under the illumination of a 1550 nm laser, with electrode A1 as the source and electrode B1 as the drain.

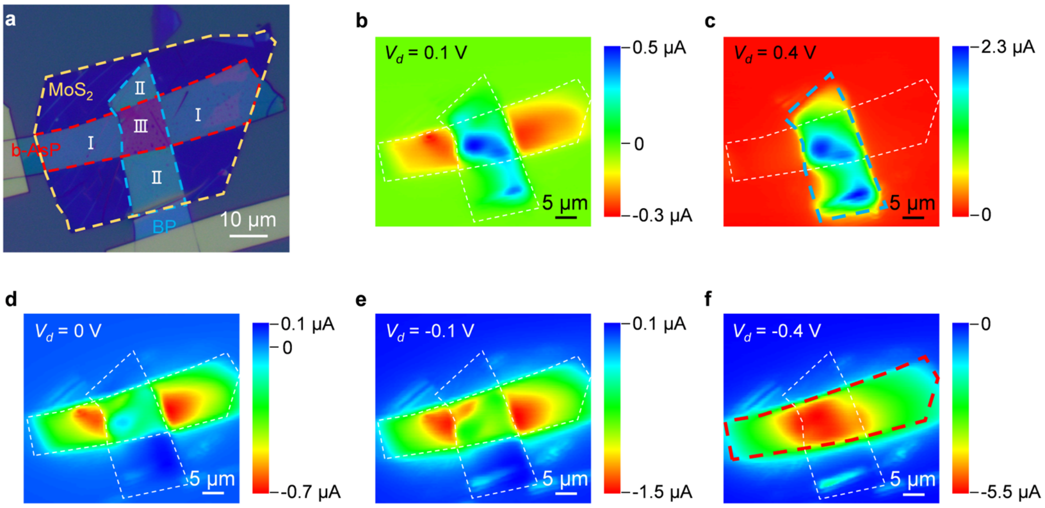

Supplementary Fig. 6 | The voltage-addressing behavior of b-AsP/MoS_2/bP pBp photodetector. a, Optical microscope image of the device. The contours of b-AsP, MoS_2, and bP highlighted by red, orange, and blue dashed lines, respectively. **b-f**, Photocurrent mapping images of device 2 at bias voltages of 0.4 V, 0.1 V, 0 V, - 0.1 V, and - 0.4 V, respectively. These measurements were performed under the illumination of an 830 nm laser.

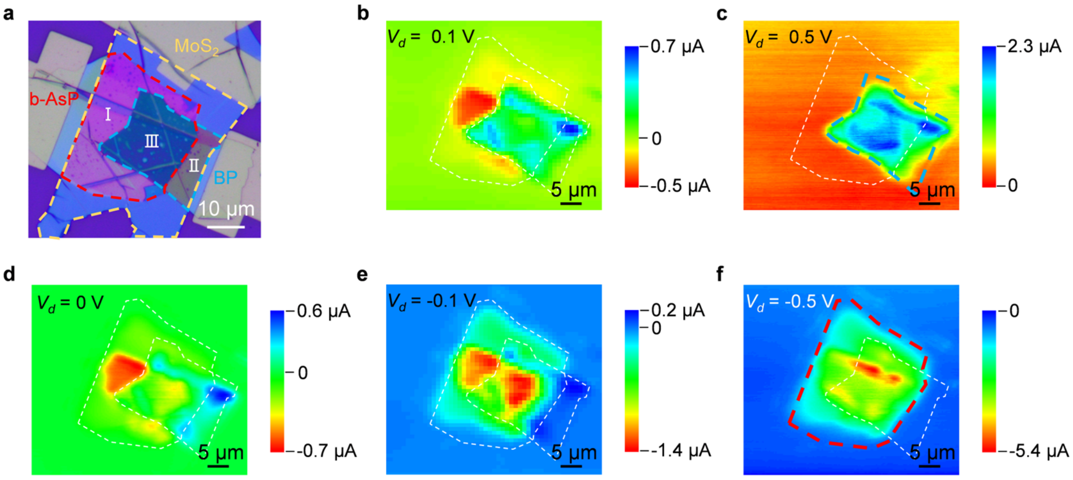

Supplementary Fig. 7 | The voltage-addressing behavior of b-AsP/MoS_2/bP pBp photodetector. a, Optical microscope image of the device. The contours of b-AsP, MoS_2, and bP highlighted by red, orange, and blue dashed lines, respectively. **b-f**, Photocurrent mapping images of device 3 at bias voltages of 0.5 V, 0.1 V, 0 V, - 0.1 V, and - 0.5 V, respectively. These measurements were performed under the illumination of an 830 nm laser.

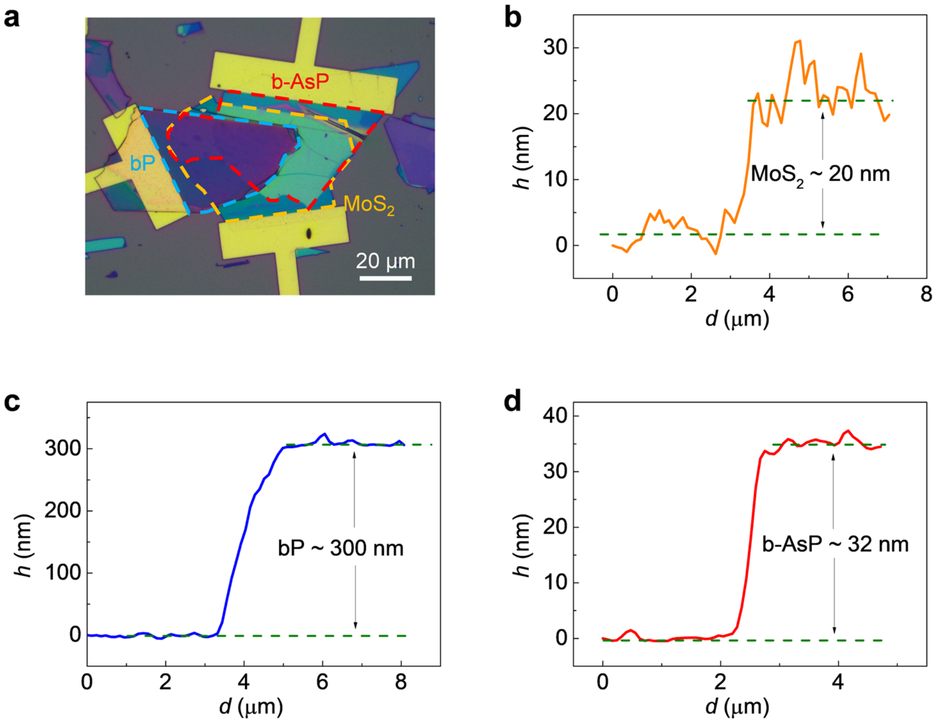

Supplementary Fig. 8 | Materials Thickness Characterization. a, The optical photo of device 4. The contours of b-AsP, MoS_2, and bP highlighted by red, orange, and blue dashed lines, respectively. **b-d**, Height profiles of MoS_2, bP, and b-AsP flakes, respectively. The corresponding thicknesses are 20, 300, and 32 nm, respectively.

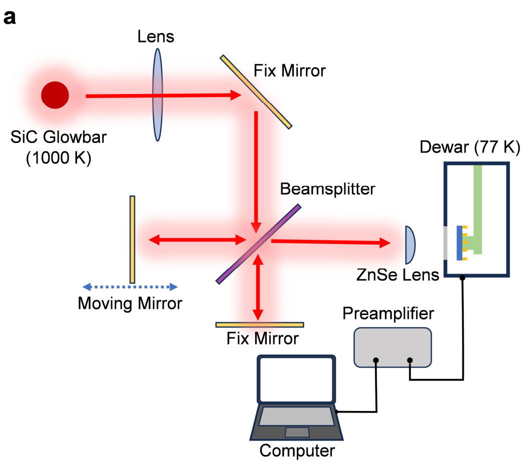

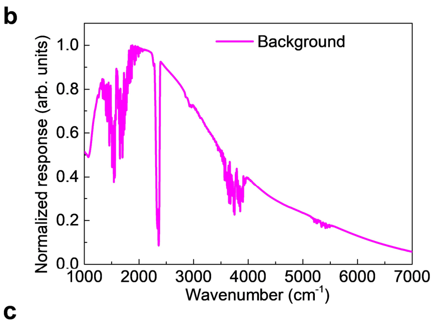

c

Incident light	Signal 1	Signal 2	Signal 2-Signal1
MWIR1	√	√	= 0
MWIR2	×	√	> 0

Supplementary Fig. 9 | Setup of Fourier transform infrared spectrometer (FTIR) test system. a, Schematic of measurement setup used to measure the spectral of the incident light on the device. **b**, Relative response spectrum of the background obtained by the internal (DTGS) photodetector. **c**, Spectral resolution dependent on the difference value between the two signals.

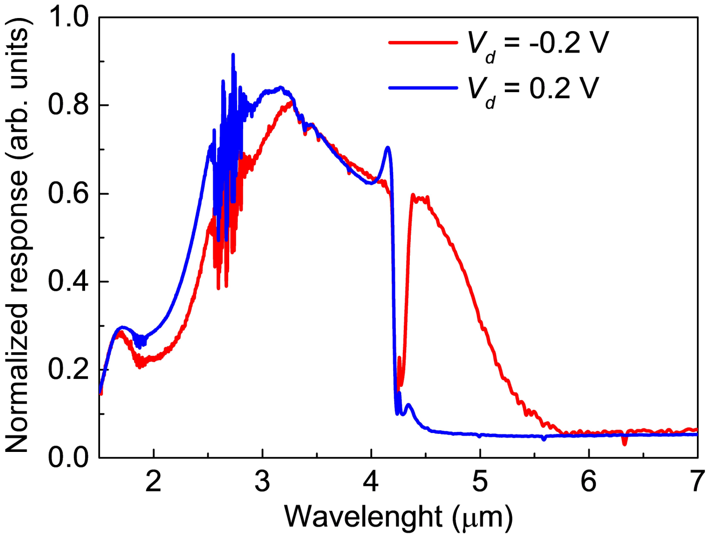

Supplementary Fig. 10 | FTIR response spectrum with non-polarized source of the device.

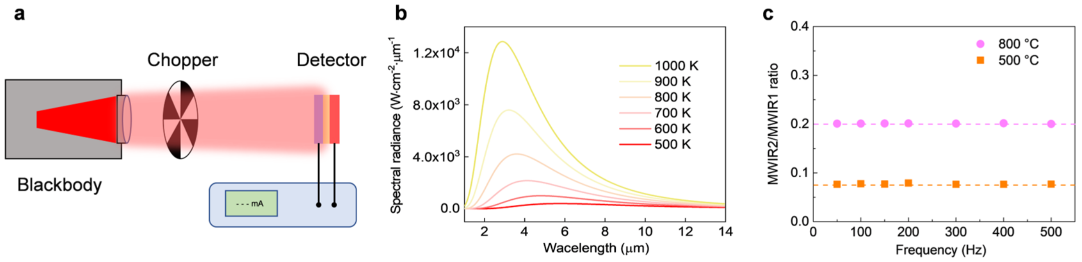

Supplementary Fig. 11 | Blackbody radiation measurements. a, Schematic of the setup of the blackbody test system for the blackbody measurements and colorimetric temperature measurements. **b**, Blackbody radiation spectra at different temperatures calculated according to Planck's law. **c**, MWIR2/MWIR1 ratio depends on the operation frequency under different blackbody temperatures.

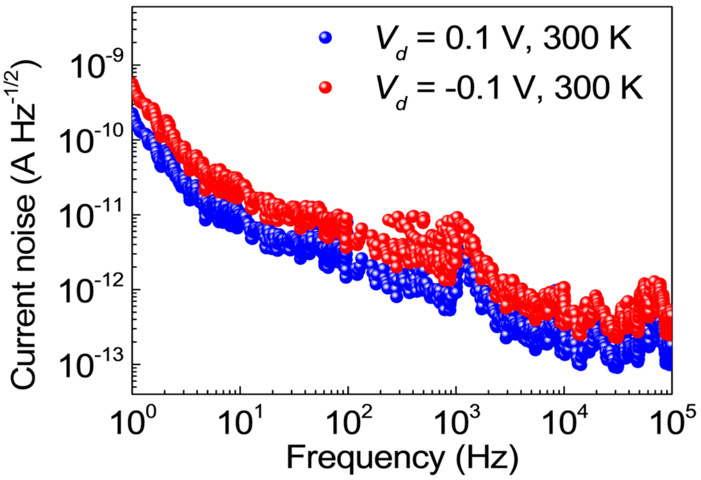

Supplementary Fig. 12 | Noise spectrum measured for the device at room temperature.

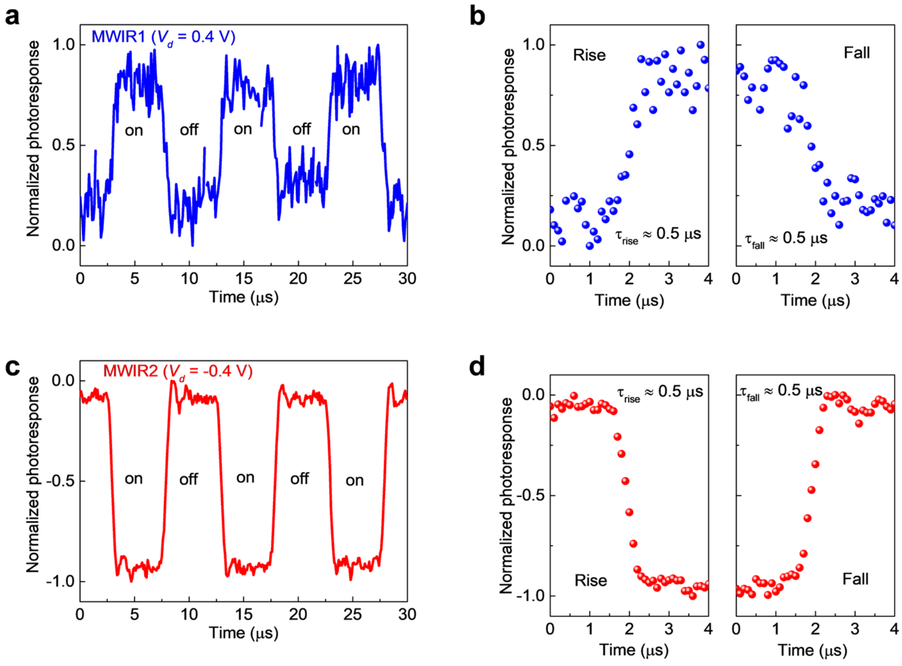

Supplementary Fig. 13 | Time-resolved photocurrent of the MWIR1 channel and MWIR2 channel under modulated laser with a wavelength of 1550 nm. a, The time response curve of the photodetector at V_d = 0.4 V. **b**, Time-resolved response of the photodetector under at V_d = 0.4 V. **c**, The time response curve of the photodetector at V_d = -0.4 V. **d**, Time-resolved response of the photodetector under at V_d = -0.4 V.

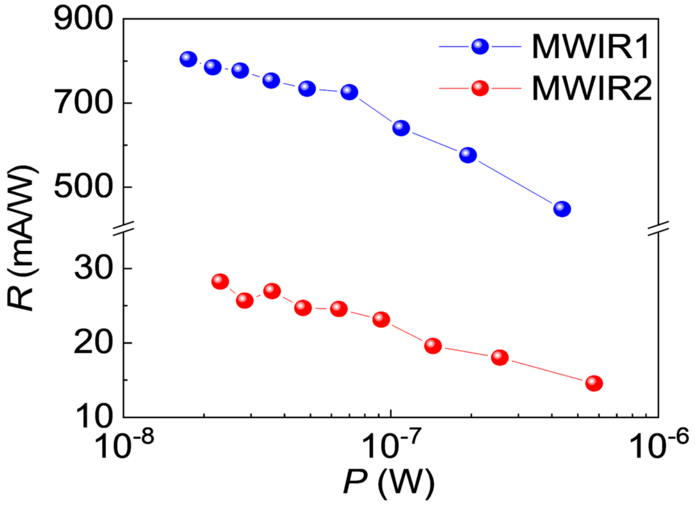

Supplementary Fig. 14 | The relationship between the responsivity and blackbody radiation power.
